# A case report of hemophagocytic syndrome induced by *Brucella melitensis* biovar 3

**DOI:** 10.3389/fimmu.2025.1695579

**Published:** 2025-11-21

**Authors:** Chao Wei, Huan Zhang, Jing Chen, Xiu Gu, Mengwei Tong, Yang Zhou, Minghui Yun, Kaiting Zhang, Tao Chen, Songsong Xie

**Affiliations:** 1 National Health Commission (NHC) Key Laboratory of Prevention and Treatment of Central Asia High Incidence Diseases, The First Affiliated Hospital of Shihezi University, Shihezi, China; 2 School of Animal Science and Technology, Shihezi University, Shihezi, China; 3 The People’s Hospital of Yining, Yining, China; 4 State Key Laboratory for Diagnosis and Treatment of Severe Zoonotic Infectious Diseases, Wuhan, China; 5 Department and Institute of Infectious Disease, Tongji Hospital, Tongji Medical College, Huazhong University of Science and Technology, Wuhan, China

**Keywords:** hemophagocytic syndrome (HPS), *Brucella*, brucellosis, AMOS-PCR, cytopenia

## Abstract

Hemophagocytic syndrome (HPS) is also called hemophagocytic lymphohistiocytosis (HLH). Hemophagocytic syndrome caused by *Brucella* infection is a rare and life-threatening complication. We report a case of a 55-year-old female farmer from China, whose symptoms included fever, pancytopenia, and liver damage. Early on, we identified the phenomenon of hemophagocytosis through blood culture and bone marrow examination, thereby confirming the case. The pathogen was precisely identified as *Brucella melitensis* Biovar 3 using AMOS-PCR technology and a systematic evolutionary analysis of the IS711 sequence, which was highly homologous to a strain of badger isolated from the same area previously. This provided molecular evidence for the potential animal-to-human transmission chain from wild animals. The patient received combined treatment with anti-infective drugs (doxycycline and rifampicin), corticosteroids, and intravenous immunoglobulin, and then followed a stepwise dose reduction treatment plan. After discharge, we conducted personalized follow-up management for the patient. This case highlights the potentially fatal complications that brucellosis can cause — hemophagocytic syndrome — and it is particularly common among individuals in high-risk occupations. For patients with brucellosis accompanied by unexplained blood cell reduction and abnormal liver function, a bone marrow puncture examination should be conducted as soon as possible. In the subsequent treatment, a combined treatment plan of “antibiotic therapy plus immunomodulation” can be adopted. Furthermore, it highlights the emerging zoonotic threat posed by *B. melitensis* biovar 3 in endemic areas.

## 1 Introduction

Brucellosis, a zoonotic infectious disease, is caused by the bacterium *Brucella*. Human infection with brucellosis primarily arises from exposure to cattle, sheep, dairy products, and excreta contaminated with *Brucella* (1). In livestock farms, cats and dogs can play a pivotal role in the dissemination and prevalence of brucellosis, serving as potential vectors and asymptomatic carriers (2). Brucellosis is prevalent in China, especially in many regions where animal husbandry is well-developed, namely, Xinjiang, Inner Mongolia, and Qinghai. The clinical manifestations of brucellosis encompass fever, hyperhidrosis, fatigue, and muscle pain, among others. Infection with *Brucella* can lead to complications affecting various bodily systems, with osteoarthritis being the most frequently encountered complication in clinical settings. However, the hemophagocytic syndrome (HPS) caused by the *B. melitensis* biovar 3 is rare (3–5). This study describes a patient who lives in Xinjiang and experienced brucellosis-associated hemophagocytic syndrome and presents a pertinent literature review.

## 2 Case presentation

A 55-year-old female without preexisting diseases began to experience upper abdominal pain in early January 2024, mainly in the middle and upper abdomen, accompanied by nausea, intermittent fever, and the highest body temperature of 39.9°C. On February 25, 2024, the patient was sent to the First Affiliated Hospital of Shihezi University due to the deterioration of the aforementioned symptoms. Initial laboratory examinations disclosed notable inflammatory and hematologic irregularities in line with a severe systemic infection (**Table 1**). Systemic inflammation was manifested by elevated C-reactive protein (39.83 mg/L), interleukin-6 (58.57 pg/mL), and procalcitonin (0.25 ng/mL). Liver function assessments indicated hepatic involvement, with substantial elevations in aspartate aminotransferase (342.5 U/L), alanine aminotransferase (120.2 U/L), alkaline phosphatase (188.1 U/L), and glutamyl transpeptidase (110.0 U/L), accompanied by hypoalbuminemia (27.2 g/L). Markedly elevated levels of lactate dehydrogenase (1072.0 U/L) and hydroxybutyrate dehydrogenase (733.0 U/L) implied extensive cellular damage. Coagulopathy was suggested by an elevated D-dimer level (5.05 mg/L). Moreover, a complete blood count revealed pancytopenia (**Table 2**), a crucial characteristic that spurred further exploration for hemophagocytic syndrome. Abdominal ultrasound showed gallbladder wall thickening. Pleural effusion color ultrasound indicated bilateral pleural effusion, with a depth of 3.2 cm on the right and 1.6 cm on the left. Abdominal CT scans revealed a cyst in the upper left outer lobe of the liver, cholecystitis, gallbladder socket effusion, splenomegaly, and fluid accumulation in the abdominal cavity and pelvis. The electrocardiogram and cardiac color ultrasound showed no abnormalities. According to the results from the clinical check, the treatment regimen encompassed the administration of Ceftriaxone (2 g q12h i.v.) for infection control, Monoammonium Cysteine Sodium Chloride Injection, and Polyene Phosphatidylcholine Injection, both aimed at enhancing liver function therapy.

**Table 1:** Summary of the laboratory tests in the patient.

Laboratory testing category	Testing items	Patient outcome	Reference range	Clinical significance
Inflammatory indicators	C-reactive protein	39.83mg/L	<6 mg/L	A significant increase suggests acute systemic inflammation or infection
	White blood cell count	1.9 × 10^9^/L	3.5-9.5 × 10^9^/L	A decrease in white blood cells indicates bone marrow suppression or immune damage, increasing the risk of infection
	Interleukin-6	58.57 pg/mL	< 7 pg/mL	A significant increase suggests a strong cytokine storm, which is associated with HPS.
	Procalcitonin	0.25 ng/mL	< 0.05 ng/mL	A mild increase indicates a bacterial infection
Liver function	Alanine aminotransferase	120.2 U/L	7–40 U/L	Elevated levels suggest liver cell damage
	Aspartate aminotransferase	342.5.0 U/L	13–35 U/L	A significant increase indicates severe liver cell damage or more extensive cell damage
	Alkaline phosphatase	188.1 U/L	38–126 U/L	Elevated levels suggest involvement of the biliary system or invasive diseases within the liver
	Glutamyl transpeptidase	110.0 U/L	7–45 U/L	A significant increase further supports the involvement of the hepatobiliary system.
	Albumin	23.3 g/L	40–55 g/L	Severe hypoproteinemia suggests malnutrition, impaired liver synthetic function or chronic wasting disease
Cell damage/metabolism	Lactate dehydrogenase	830.0 U/L	120–246 U/L	Extremely elevated, indicating extensive cellular damage (such as hemolysis, lymphoma, or tissue damage related to HPS).
	Hydroxybutyrate dehydrogenase	733.0 U/L	72–182 U/L	Increase, along with LDH, supports extensive tissue cell damage.
	Uric acid	80.0 μmol/L	155-357 μmol/L	It is relatively low. In severe systemic diseases, it may be caused by malnutrition or excessive consumption.
Coagulation function	D-dimer	5.05 mg/L	< 0.55 mg/L	A significant increase suggests secondary hyperfibrinolysis, which is commonly seen in infections, inflammations or coagulation disorders

**Table 2:** Follow-up records of blood tests in patients.

Date	WBC, ×10^9^/L	Hb, g/L	PLT, ×10^9^/L	RBC, ×10^12^/L	N, %	L, %	APTT, sec	PT, sec	FIB, g/L	FER, ng/mL	TG
02/26	1.9	90	76	3.48	59.9	35.5	41.3	14	1.34	>2000.00	ND
02/28	2.6	84	49	3.18	49.0	43.9	ND	ND	ND	ND	ND
03/05	2.3	77	56	2.98	69.0	28.0	42.9	13.8	1.46	>2000.00	1.23
03/09	2.8	76	153	2.95	42.0	46.0	26.9	9.9	2.03	ND	ND
03/20	5.1	101	354	3.74	52.3	41.6	ND	ND	ND	ND	ND
03/27	3.7	105	268	3.87	46.4	44.5	ND	ND	ND	ND	ND
04/11	6.0	109	183	3.84	70.0	24.4	ND	ND	ND	ND	ND
04/24	2.8	118	189	4.11	41.2	47.0	ND	ND	ND	ND	ND
05/08	2.5	124	191	4.23	27.4	58.1	ND	ND	ND	ND	ND
05/22	2.6	121	163	4.05	29.8	53.8	ND	ND	ND	ND	ND
06/05	2.9	129	162	4.29	40.1	48.3	ND	ND	ND	ND	ND

WBC, white blood cell; Hb, hemoglobin; PLT, platelets; RBC, red blood cell; N, neutrophils; L, lymphocytes; APTT, activated partial thromboplastin time; PT, prothrombin time; FIB, fibrinogen; FER, ferritin; TG, triglyceride; ND, not detect.

**Figure 1:**
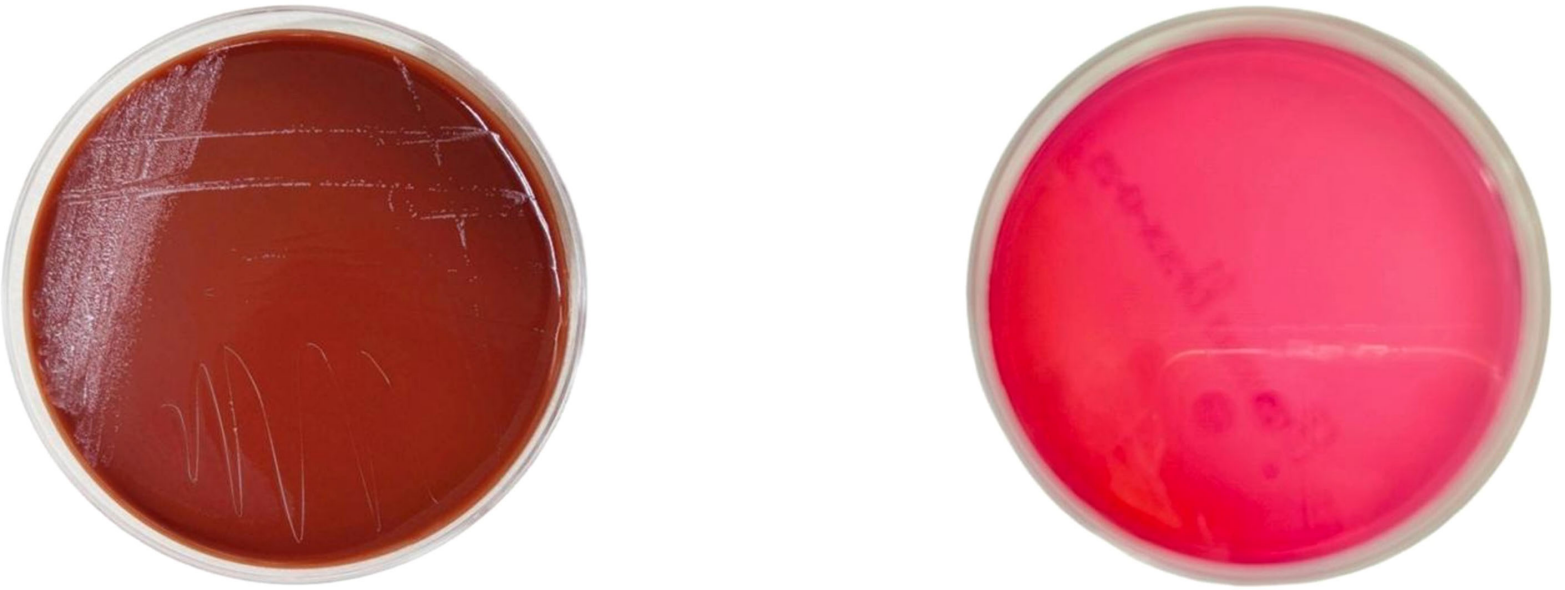
Patient’s blood culture positive result chart.

Because the patient had a history of exposure to cattle and sheep, and lived in an area where brucellosis is endemic. To make a clear diagnosis, before administering antibiotics on February 28th, when the patient was admitted to the hospital, we strictly followed the aseptic operation procedures to collect blood samples and inoculate them into BACTEC blood culture bottles (see **Supplementary Methods**). On March 4th, the blood culture results were positive. The pathogen detected through AMOS-PCR (as shown in **Tables 3**, **4**) was the *Brucella melitensis* (**Figure 1**) According to the results of the pathogen, the treatment regimen was adjusted to ceftriaxone (2 g/12h IV infusion), rifampicin (0.6 g/d, oral), and doxycycline (100 mg, twice/d, oral) for targeted anti-infective therapy.

**Table 3:** Primers and sequences for *Abortus, Melitensis, Ovis*, and *Suis*-PCR (AMOS-PCR).

Primer names	Sequences (5’-3’)	Size, bp
*B*.*abortus*-IS711- primer –F (BA)	GACGAACGGAATTTTTCCAATCCC	498
*B.abortus*-IS711- primer -R	TGCCGATCACTTAAGGGCCTTCAT	
*B. melitensis*- IS711- primer -F (BM)	AAATCGCGTCCTTGCTGGTCTGA	731
*B. melitensis*- IS711- primer -R	TGCCGATCACTTAAGGGCCTTCAT	
*B. ovis*- IS711- primer -F (BO)	CGGGTTCTGGCACCATCGTCG	976
*B. ovis*- IS711- primer -R	TGCCGATCACTTAAGGGCCTTCAT	
*B. suis*- IS711- primer -F (BS)	GCGCGGTTTTCTGAAGGTGGTTCAGG	285
*B. suis*- IS711- primer -R	TGCCGATCACTTAAGGGCCTTCAT	

**Table 4:** Reaction conditions for AMOS-PCR.

PCR Procedure	Temperature, °C	Time	Cycles
Predevaluation	95	5min	1
Denature	94	40s	35
Annealing	61	1min	35
Extension	72	3min	35
Final extension	72	10min	1
Thermal insulation	4	1h	

**Figure 2:**
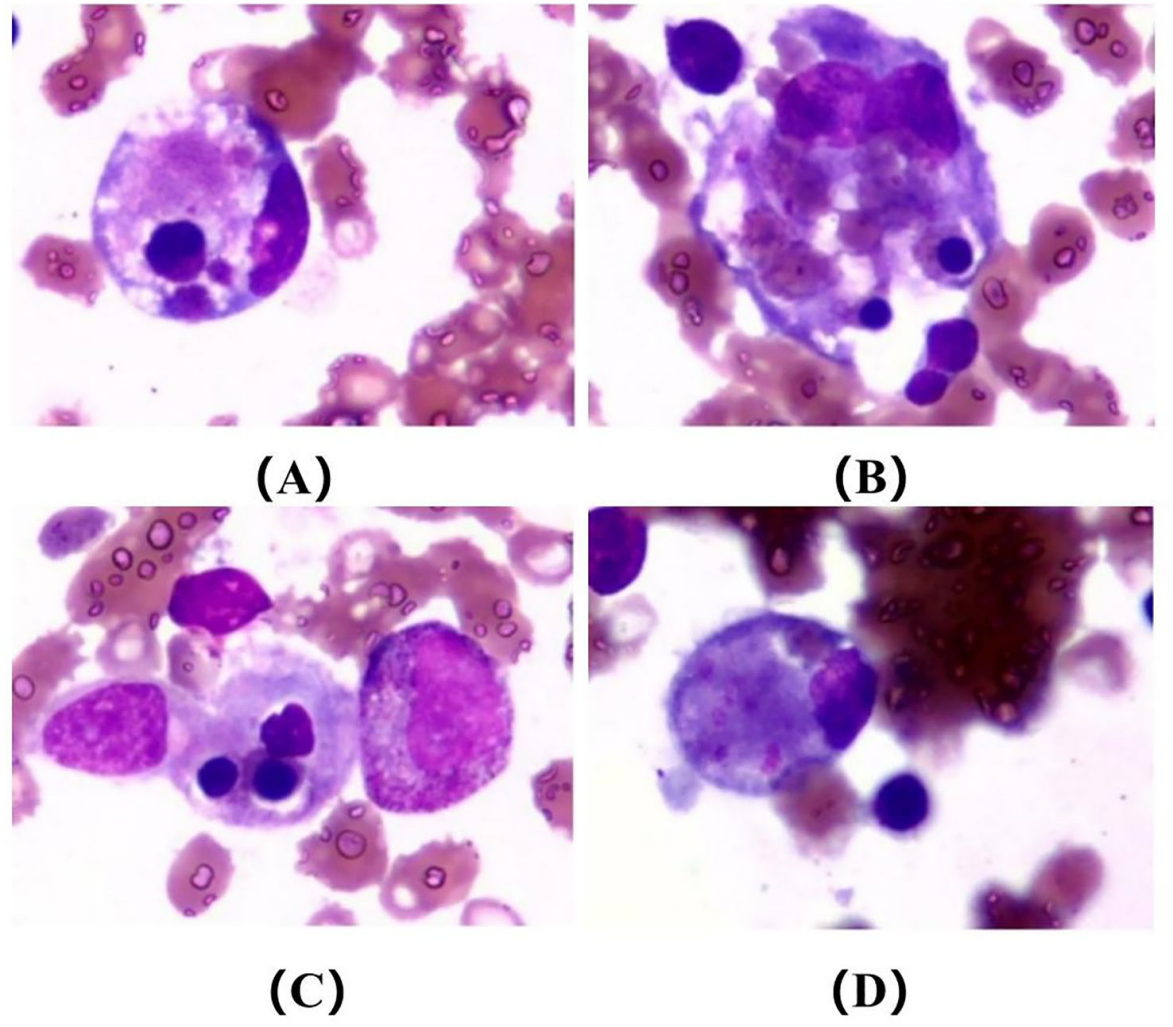
Depicts a bone marrow smear, exhibiting characteristics of hyperplastic bone marrow. Histiocytes and hemophagocytes comprise approximately 3% of the cellular composition, with a concurrent presence of thrombocytopenia. Original magnification×1,000.

Furthermore, given the patient’s progressive decrease in complete blood count, the clinical team decided to perform a bone marrow aspiration (see **Supplementary Methods**) on February 28th to identify the cause. Results of a bone marrow smear on March 5th revealed hyperplastic bone marrow, with 3% histiocytes and hemophagocytes, along with thrombocytopenia (**Figure 2**). In combination with the findings of hemophagocytosis in the bone marrow and based on HLH-2004 criteria, the patient was finally diagnosed with brucellosis-associated hemophagocytic syndrome (**Table 5**). Therefore, based on the original anti-infective regimen, intravenous dexamethasone (15 mg, once daily) was immediately added for immunosuppressive therapy. Two days after the initiation of the protocol, her temperature normalized. Following the aforementioned treatment, on March 10th the patient was discharged. The discharge plan was: 1) Doxycycline (100 mg, twice daily, orally), Rifampin (0.6 g, once daily, orally), and Levofloxacin (0.5 g, once daily, orally) (for brucellosis treatment). 2) Bicyclic alcohol tablets (25 mg, three times daily, orally) and Glucolactone tablets (0.2 g, three times daily, orally) (for liver protection treatment). 3) Prednisone acetate tablets (45 mg, once daily, orally) (for Hemophagocytic syndrome treatment).

**Table 5:** The patient was diagnosed according to the HLH-04 standard.

Diagnostic criteria for HLH used in the HLH-2004 trial*	This case
**A. Molecular diagnosis consistent with HLH: pathologic mutations of PRF1,UNC13D, Munc18-2, Rab27a, STX11, SH2D1A, or BIRC4**	**ND**
**B. Five of the eight criteria listed below are fulfilled:**	
** 1. Fever ≥ 38.5 °C**	**Y**
** 2. Splenomegaly**	**Y**
** 3. Cytopenias (affecting at least 2 of 3 lineages in the peripheral blood):** ①Hemoglobin< 90 g/L (in infants < 4 weeks: hemoglobin < 100 g/L); ②Platelets < 100 × 10^9^/L; ③Neutrophils < 1.0 × 10^9^ and not caused by reduced bone marrow hematopoietic function.	**Y**
** 4. Hypertriglyceridemia (fasting,>265 mg/dL) and/or hypofibrinogenemia (< 150 mg/dL)**	**Y**
** 5. Hemophagocytosis in bone marrow, spleen, lymph nodes, or liver**	**Y**
** 6. Low or absent NK-cell activity**	**ND**
** 7. Ferritin > 500 ng/mL**	**Y**
** 8. Elevated sCD25 (a-chain of slL-2 receptor)**	**ND**

* The currently recognized diagnostic criteria for HLH were revised by the International Society for Cellular Therapy in 2004. HLH can be diagnosed if either criterion A or B is met.

ND, Not detected; Y, Yes.

**Figure 3:**
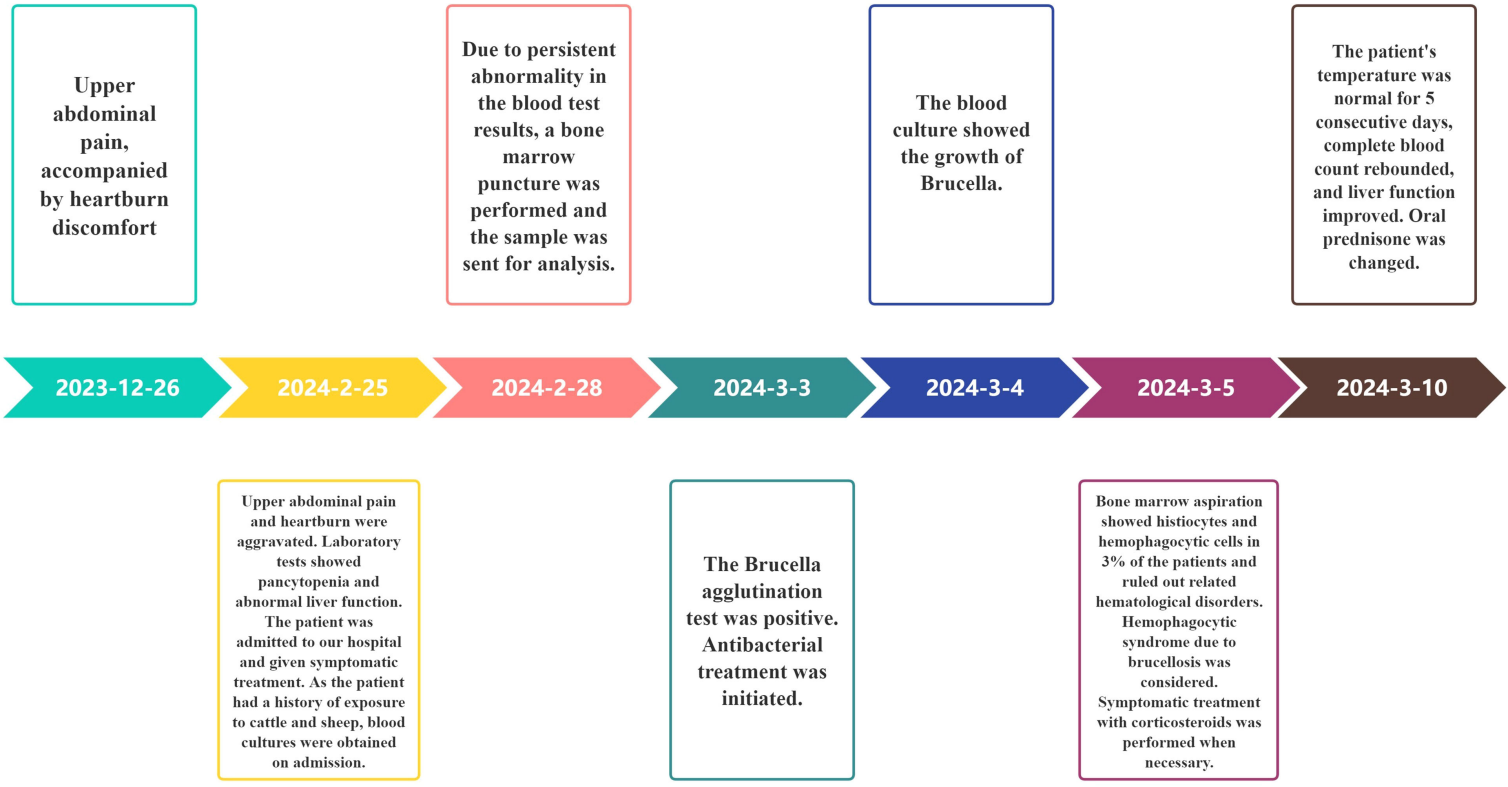
The onset, progression, diagnosis, and treatment of this patient.

**Figure 4:**
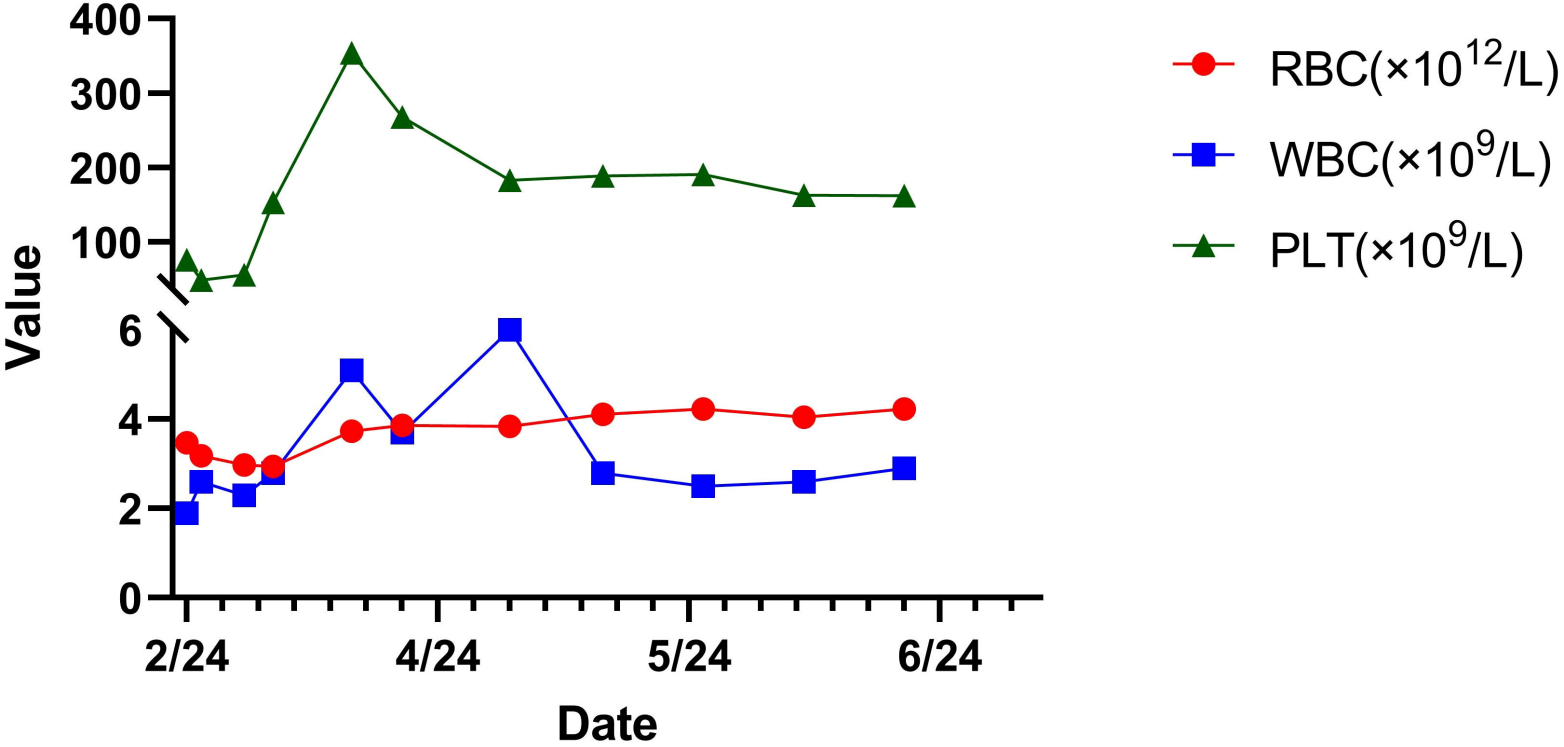
Graphical representation of the associated trends in white blood cell count, platelet count, and red blood cell count in this patient.

**Figure 5:**
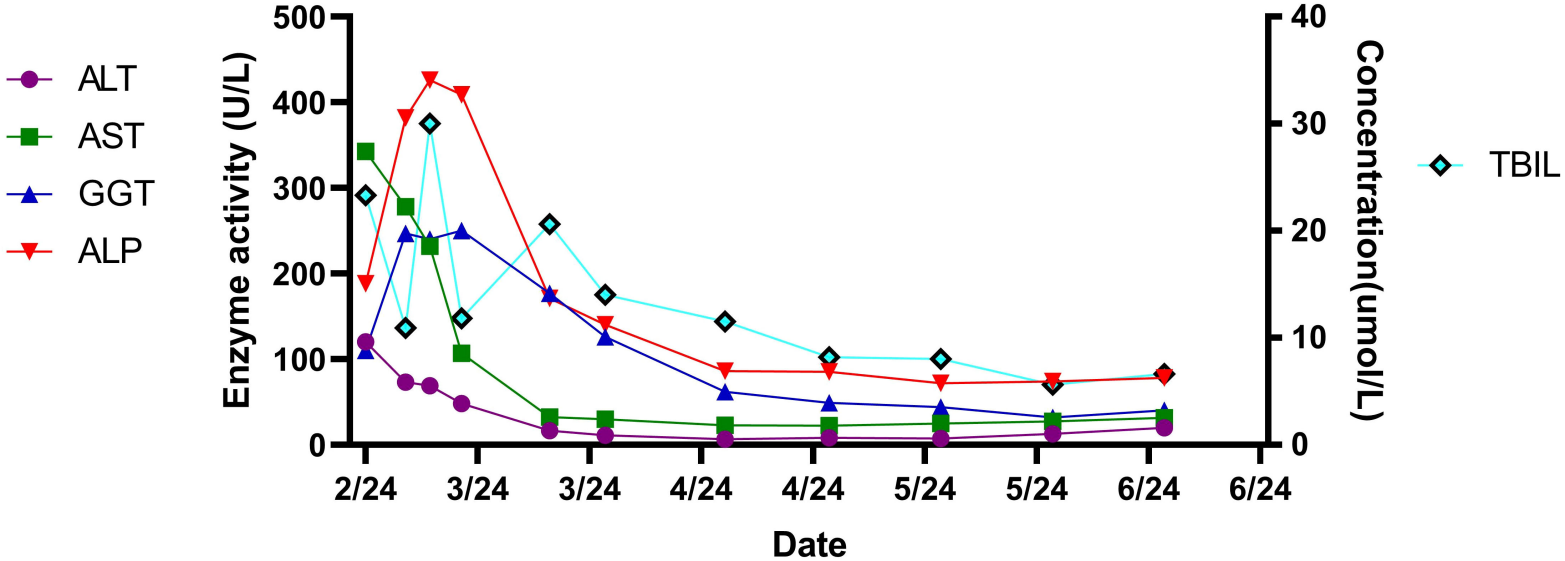
The trend of the patient’s liver function-related indicators.

**Figure 6:**
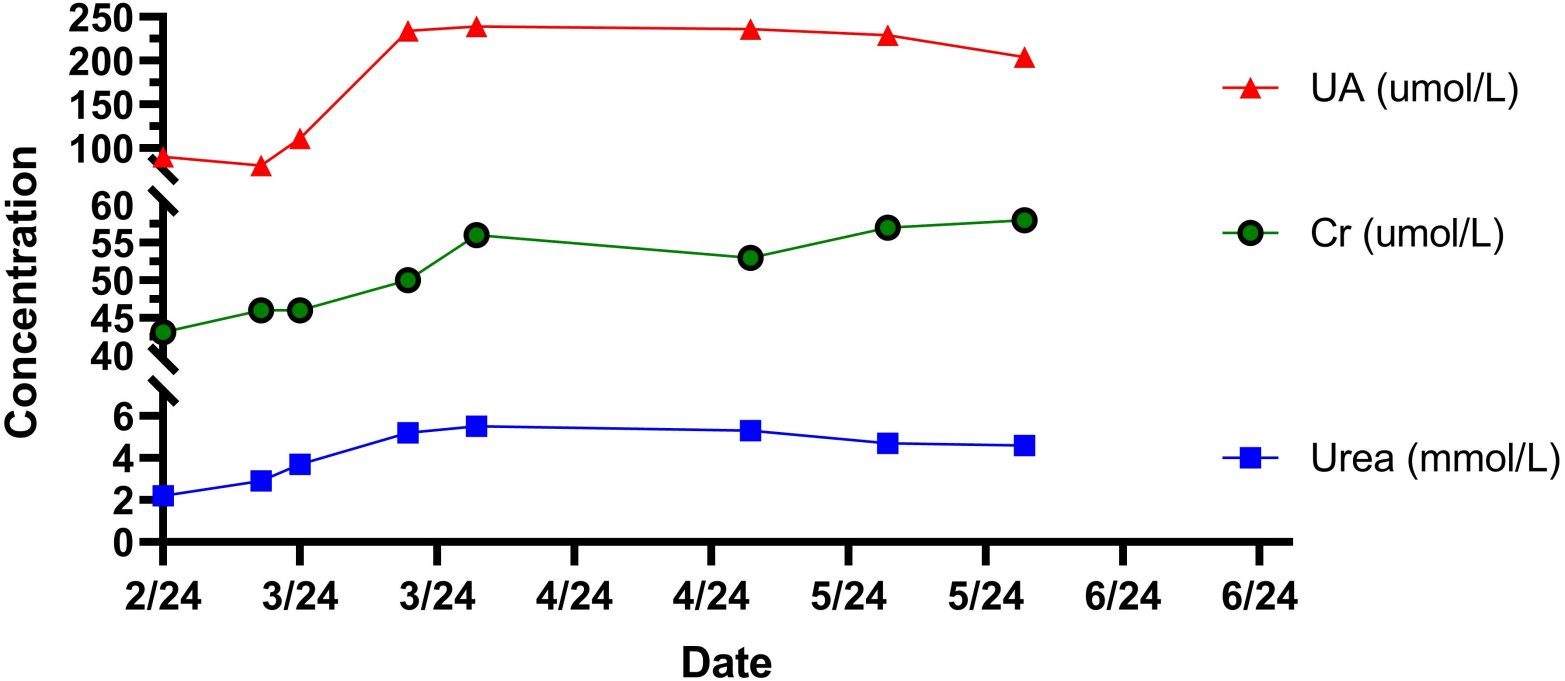
The trend of the patient’s renal function-related indicators.

Post-discharge, the patient was followed up in March, April, May, and June. The patient did not experience abdominal pain, fever, or other discomfort. The resolution of the pleural effusion was confirmed by ultrasound, and red blood cells, hemoglobin, and platelets returned to normal levels. However, the white blood cell count (2.9×10^9^/L) was lower than normal. Levofloxacin was discontinued after six weeks of treatment, while Rifampin and Doxycycline were used for a total of 16 weeks. Concurrently, the oral hormone underwent a gradual reduction in dosage and was ultimately discontinued within an overall treatment duration of 11 weeks, until the number of white blood cells returned to the normal level (**Table 2****).** The development, diagnosis, and treatment of this disease are shown in **Figure 3**. During the treatment of this disease, the trends of changes in blood cells, liver function, and kidney function can be observed in **Figures 4**–**6**.

## 3 Results

### 3.1 Pathogen identification

**Figure 7:**
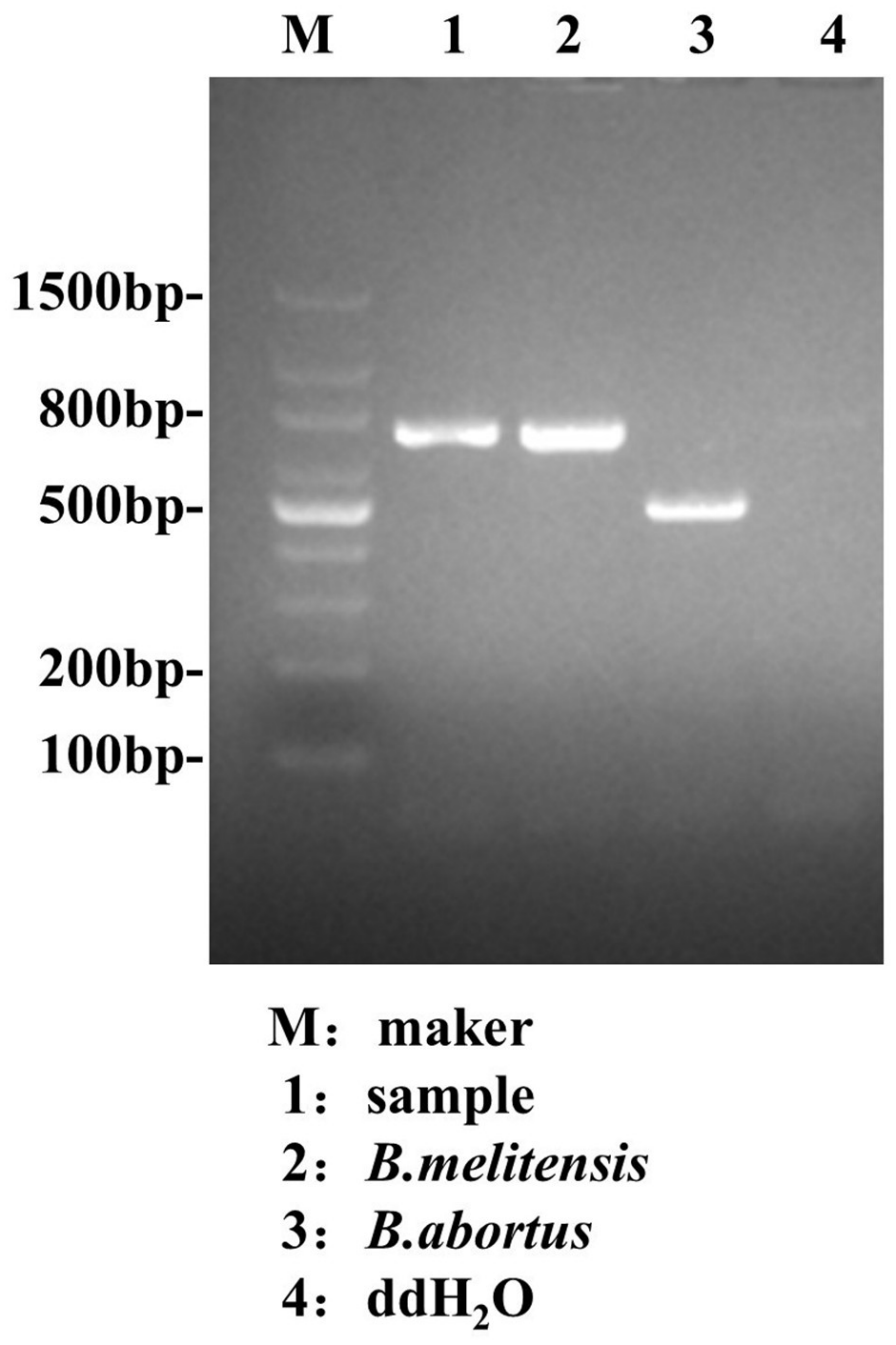
Results of the identification of this *Brucella* by AMOS-PCR.

**Figure 8:**
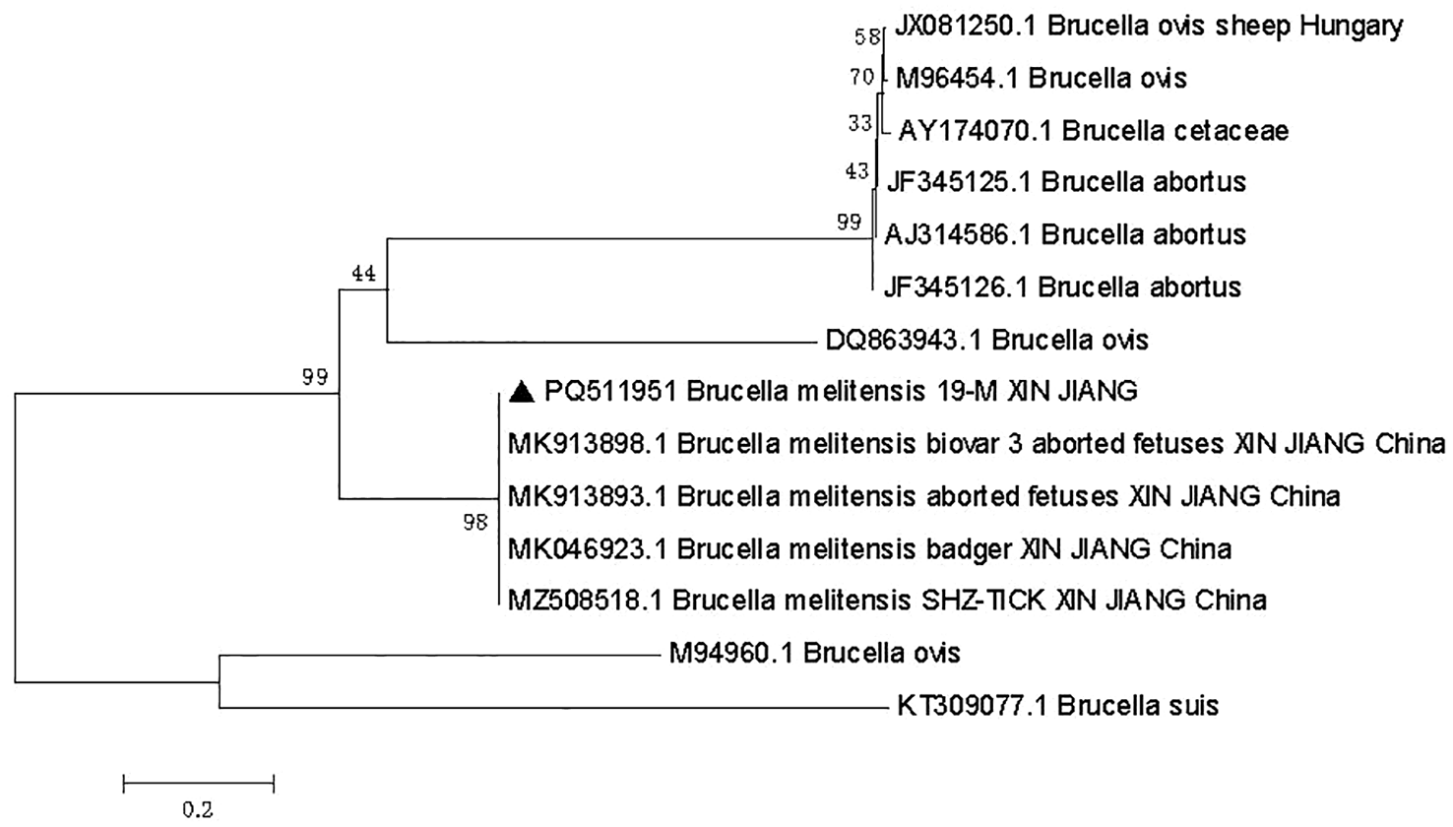
Phylogenetic tree of the IS711 tandem sequence of *Brucella melitensis* (•) isolated from the patient’s blood. According to the adjacency method (NJ; 500 bootstrap replicates with MEGA7) and maximum likelihood (ML, 1000 bootstrap replicates) analyses. Scale bars represent the inferred substitutions for each nucleotide site.

The PCR products (**Figure 7**) were then sequenced, and a phylogenetic tree was constructed based on the 731 bp sequence of the IS711 duplicate. The analysis of the research results indicated that the *Brucella* isolated in this study exhibited a high degree of similarity to *Brucella melitensis* isolated from a badger in Xinjiang, China (**Figure 8**).

### 3.2 Literature review

Based on brucellosis and hemophagocytic lymphohistiocytosis, the related cases published by PubMed in the last 20 years were reviewed. The diagnosis of brucellosis complicated with hemophagocytic syndrome is mainly based on blood culture and bone marrow puncture, symptoms, and laboratory examination. Most studies only control the disease by actively treating the primary infection, and a few studies adopt the method of treating the primary infection and gamma globulin or hormone shock (**Table 6**).

**Table 6:** Literature review.

Authors	Age/ sex ^a^	Career/contact history	Symptoms/signs	Confirmed exams		Therapeutic regimen	
				*Brucella* ^b^	BMA ^c^	Anti-*Brucella*^d^	HPS ^e^
HEYDARI A A, et al. (6)	45/F	Farmer/	fever, hepatosplenomegaly, jaundice, rash, and tachypnea	WAT	Y	DOX plus GM	HC (200mg/day) for 3 days
ERDURAN E, et al. (7)	8/M	-/milk products	weight loss, arthralgia, prolonged fever, sweating, and fatigue	SAT (1:640)	Y	RFP (15 mg/kg/day) for 6 weeks and TMP-SMX (10 mg/kg/day) for 6 weeks.	-
ERDEM E, et al. (8)	12/M	-	fever, loss of appetite, and headache,	WAT (1:2560)	Y	DOX 200 mg/day for 6 weeks and nous GM 5 mg/kg/day for 2 weeks	-
YAMAN Y, et al. (9)	4/M	-/fresh milk products	fever, chills, and fatigue	Coombs 1:320	Y	RFP (10 mg/kg/day), GM, and DOX for 6 weeks	IVIG with a dose of 1 g/kg/day for two days
	16/M	-/non pasteurized milk products	fever, chills, and fatigue	Coombs 1:640 BC	Y	RFP (600 mg/day) and DOX (200 mg/day) for 6 weeks	IVIG with a dose of 1 g/kg/day for two days
	8/M	-/fresh milk products	fever, fatigue, sweating, and abdominal pain	SAT (1:2,560) BC	Y	RFP (600 mg/day) and DOX (200 mg/day) for 6 weeks	IVIG with a dose of 1 g/kg/day for two days
AYDN S, et al. (10)	73/M	Farmer/-	Fever, loss of appetite, and back pain	RBT: Positive Coombs: 1/1280 BC	–	DOX and RFP for 6 weeks	-
JIN J G (11)	53/M	-/delivering lamb without proper protection	fatigue, arthralgia, and fever	RBT: Positive SAT 1: 400 BC	Y	DOX (200 mg/day), and RFP (600 mg/day) for 12 weeks; FLU (50 mg/day IV for 3 days)	DEX (10 mg/m^2^/day), and gradually decreasing
AL NOUMANI J, et al. (12)	50/F	Herdsman/goats	fever, chills, drenching night sweats, malaise and weight loss	BC	Y	GM (5 mg/kg) for seven days, DOX 100 mg tid, for 3weeks and oral CIP 750 mg tid, for 3weeks. Oral administration for 3 weeks after discharge.	-
ZHANG Y, et al. (13)	41/F	-/recent trip to the area with well-developed animal husbandry	fever, loss of appetite, and pancytopenia	BC	Y	DOX and RFP	
PARK D, et al. (14)	60/M	-/-	fevers, chills, night sweats, and weight loss	BC SAT (>1:1280)	Y	DOX 100 mg twice daily (BID) and RFP 100 mg BID for six weeks.	DEX 10 mg/m^2^, and gradually decreasing

^a^ M, male; F, female.

^b^ WAT, Wright’s serum agglutination; BC, blood culture; RBT, Rose Bengal plate.

^c^ Y, yes.

^D^ DOX, doxycycline; GM, gentamicin; RFP, rifampin; TMP-SMX, trimethoprim-sulfamethoxazole; FLU, Fludarabine; CIP, ciprofloxacin.

^e^ HC, hydrocortisone; IVIG, immunoglobulin; DEX, dexamethasone.

## 4 Discussion

This case reports a patient with brucellosis causing hemophagocytic syndrome, whose clinical presentation included fever, splenomegaly, hemopenia, hypertriglyceridemia, hypofibrinogenemia, and elevated serum ferritin, and hemophagocytosis was seen by bone marrow aspiration. The diagnosis of brucellosis causing hemophagocytic syndrome was confirmed by a detailed history taking, physical examination, and key ancillary investigations, and was finalized based on blood cultures and HLH-2004 guidelines. In terms of treatment, we used anti-*Brucella* treatment and treatment for hemophagocytic syndrome, and adjusted the treatment plan according to the patient’s condition. The treatment results showed that the patient’s symptoms were significantly relieved, and the disease was effectively controlled. Brucellosis-induced phagocytic syndrome mainly occurs in the acute phase of brucellosis, and the occurrence of combined phagocytic syndrome should be alerted when clinical findings of fever, splenomegaly, or liver function abnormalities, hematocrit, elevated serum ferritin (15), and elevated sCD25 are found in brucellosis infection.

In addition, during the follow-up of this case, we noted a clinical problem of persistent leukopenia. As shown in **Table 2**, although the patient’s anemia and thrombocytopenia resolved rapidly after the initiation of antimicrobial and immunosuppressive therapy, the white-cell counts, particularly neutrophils, remained at the lower limit of normal throughout the months-long follow-up period. This phenomenon is more likely to reflect a delayed recovery of bone marrow hematopoiesis after hemophagocytic lymphohistiocytosis than a simple drug side effect. Hemophagocytic syndrome strongly inhibits bone marrow hematopoietic precursor cells through multiple mechanisms, such as cytokine storm, direct hemophagocytosis, and infectious destruction. This inhibition not only leads to a rapid decline in blood cell lines but also destroys the structure and function of the bone marrow microenvironment, making the reconstruction of the microenvironment and the recovery of hematopoietic function take longer (16, 17). Immunosuppressive agents such as glucocorticoids themselves have significant myelosuppressive effects, and long-term use may also contribute to this phenomenon. This observation suggests that for survivors of brucellosis-associated HPS, the full recovery of bone marrow function may be a slower process that requires long-term hematologic monitoring. Fortunately, in this case, leukopenia caused no new infectious events and eventually normalized.

Hemophagocytic syndrome is a syndrome of excessive inflammatory response caused by abnormal activation, proliferation, and secretion of large amounts of inflammatory cytokines by lymphocytes, monocytes, and macrophages due to abnormalities in hereditary or acquired immunoregulatory functions. Secondary phagocytic syndromes, on the other hand, are often caused by a variety of triggers such as tumors, rheumatic immune diseases, and infections. The main triggers of hemophagocytic syndrome due to infections are viruses, bacteria, and fungi. Among them, *Brucella* invasion can activate the immune system, and the cytokines secreted by the immune system play a key role in the immune response. Studies have shown that the frequency of γ-interferon (IFN-γ) and tumor necrosis factor-α (TNF-α) in patients with acute-phase *Brucella* is significantly higher than that in healthy people (18). On the one hand, IFN-γ activates other cytokine pathways through the activation of macrophages, leading to a cascade response of inflammatory factors; it also activates the JAK-STAT signal pathway and activates the transcription of inflammatory factor-related genes. On the other hand, TNF-α activates the NF-κB pathway, causing increased secretion of cytokines (e.g., IL-12, IL-2, IL-1, IL-6, IL-10, granulocyte-macrophage colony-stimulating factor (GM-CSF), etc.) (19, 20). These inflammatory factors can lead to clinical signs of phagocytic syndrome, such as bone marrow suppression, lymph node enlargement, fever, and abnormal organ function (19).

Since hemophagocytic syndrome is caused by the excessive release of inflammatory mediators, the main treatment is cytokine reduction and supportive therapy. Currently, the treatment of phagocytic syndrome is based on the HLH-2004 protocol, which covers immunosuppressive regimens such as dexamethasone, cyclosporine, and etoposide. In secondary phagocytic syndromes caused by infections, the primary therapeutic goal is to remove the infectious agent, and antimicrobial or antiviral drugs are the key to treatment and should be administered at the start of therapy. In cases of high cytokine levels, a combination of high-dose intravenous immunoglobulins and steroids should also be considered. In addition, in secondary phagocytic syndromes caused by (non-viral) infections, adjuvant corticosteroids or anti-cytokine therapy targeting IL-1 or IL-6 may be used to accelerate recovery and/or improve survival (20–23).

The systems most commonly involved in brucellosis complications are the osteoarticular system, followed by the digestive, respiratory, genitourinary, cardiovascular, and neurological systems, as well as the blood and skin. Among the other hematologic diseases caused by brucellosis are those detailed in **Table 7**.

**Table 7:** Disease of the blood system caused by brucellosis.

Symptoms	Characteristic or mechanism
Anemia	Bone marrow suppression, chronic inflammatory depletion, hypersplenism or immune hemolysis
Abnormalities in white blood cells	Bone marrow suppression, immune-mediated destruction or infection directly inhibit the production of white blood cells.
Thrombocytopenia (24, 25)	Hypersplenism, myelosuppression, disseminated intravascular coagulation, direct damage to platelets by viruses and bacteria, hemophagocytosis, granulomas, and immune mediation.
Pancytopenia (25–28)	Hemophagocytosis, hypersplenism, bone marrow aplasia, bone marrow granulomas, and immune destruction
Disseminated intravascular coagulation (29)	Bacterial products such as endotoxin can cause endothelial damage or bind to platelets, allowing them to aggregate and be cleared from circulation.
Gammopathy (30)	_—_
Multiple myeloma (30)	Persistent fever, muscle weakness, loss of appetite and night sweats and anemia, polyclonal gammopathy, elevated urea and creatinine, increased ESR and CRP protein levels, and a high proportion of plasma cells (40%) observed in bone marrow puncture.

In recent years, the hemophagocytic syndrome caused by brucellosis has become an increasingly important clinical problem. Due to the lack of specificity of the clinical manifestations of brucellosis and the fact that hemophagocytic syndrome is a life-threatening inflammatory syndrome, clinicians are prone to miss and misdiagnose these diseases. Early diagnosis and timely treatment play a decisive role in saving patients’ lives and improving their prognosis.

For brucellosis-induced hemophagocytic syndrome, treatment should be devoted to controlling *Brucella* infection and suppressing excessive immune response. In the course of treatment, patients’ clinical symptoms and laboratory indicators should be closely monitored, and the treatment program should be adjusted in time.

Phylogenetic analysis based on the IS711 locus revealed that the patient’s *B. melitensis* Biovar 3 strain clustered closely with a sequence previously isolated from a badger in the same region of Xinjiang. This high homology provides compelling molecular evidence for a potential zoonotic transmission chain from wild animals to humans in this endemic area. It underscores the importance of wildlife reservoirs in the epidemiology of human brucellosis. However, it is important to acknowledge the limitations of our phylogenetic inference. Based on a single genetic marker (IS711), which, while useful for species and biovar identification, lacks the resolution of whole-genome sequencing (WGS) for precise strain tracking and robust phylogenetic conclusions. A more definitive analysis, such as core-genome SNP (Single Nucleotide Polymorphism) comparison with other human-derived *B. melitensis* genomes from across Asia, would be required to accurately determine the genotype of our isolate and its exact placement within the global *B. melitensis* population structure.

## 5 Conclusion

HPS secondary to *Brucella melitensis* infection, though rare, represents a severe and potentially fatal complication of brucellosis, particularly in individuals with occupational exposure to livestock. This case clearly demonstrates the significance of conducting early diagnosis through comprehensive microbiological, molecular, and histopathological evaluations. For cases of unexplained blood cell reduction and abnormal liver function, Brucellosis should be suspected; failure to diagnose promptly can lead to rapid deterioration of the condition. The successful treatment of this patient relied on early bone marrow puncture, as well as targeted anti-infection therapy (effective against *Brucella*) and immunomodulation (corticosteroids and intravenous immunoglobulin). This highlights the need for a comprehensive treatment strategy in the treatment of HPS.

Furthermore, phylogenetic analysis linking the isolate to a zoonotic strain from Xinjiang emphasizes the role of wildlife reservoirs in human brucellosis and calls for heightened surveillance of *Brucella* transmission dynamics in endemic regions. Public health measures should prioritize education for high-risk occupations (e.g., farmers and herders) and optimize diagnostic protocols in resource-limited settings to mitigate delays in treatment. Future research should not only explore the immunopathogenic mechanism of HPS caused by *Brucella* to optimize treatment plans and enhance therapeutic outcomes, but also incorporate high-resolution genomic monitoring of *Brucella* isolates to elucidate the transmission dynamics and the potential virulence of specific genotypes.

## Data Availability

The original contributions presented in the study are included in the article/**Supplementary Material**. Further inquiries can be directed to the corresponding authors.
