# Supplementary material for: A case report of hemophagocytic syndrome induced by Brucella melitensis biovar 3
Source: Front Immunol. 2025 Nov 21;16:1695579. doi: 10.3389/fimmu.2025.1695579 (PMC12678373; doi:10.3389/fimmu.2025.1695579)
Supplement: Supplementary file 1 [file DataSheet1.pdf]

Supplementary Methods

1. Blood culture

(1) Preparation before collecting blood culture samples

1) Personnel and Materials Preparation

①**Personnel qualifications:** The operation must be carried out by a registered nurse with at least 3 years of clinical nursing experience. Before the operation, they need to complete specialized training on brucellosis prevention and aseptic blood collection. Only after passing the assessment can they start working.

② **List of items:**

a. Sterile consumables: Disposable 20mL syringes (with 7-gauge needles), BACTEC aerobic blood culture bottles (Becton Dickinson, USA), BACTEC anaerobic blood culture bottles (same brand), sterile gauze, sterile gloves;

b. Disinfection supplies: 2% chlorhexidine gluconate - 70% isopropyl alcohol compound disinfectant solution (single-dose packaging), disinfection swabs;

c. Auxiliary supplies: Tourniquets, disposable treatment towels, labels (indicating patient name, hospital number, collection time, site), biosafety transport box (preheated to 20-25°C).

2) Patient Preparation and Assessment

①**Communication notification:** Explain to the patient and their family the purpose of blood collection (to confirm the diagnosis of brucellosis), the operation process (approximately 5-10 minutes), and the key points for cooperation (avoiding physical movement), to alleviate their anxiety, and obtain the signed informed consent form.

② **Physical assessment:**

a. Vascular assessment: The brachial vein at the elbow is the preferred site for examination. Check if the skin at the puncture site is red, swollen, damaged or scarred. If so, switch to the contralateral upper limb or the great saphenous vein of the lower limb;

b. Confirmation of underlying diseases: Ask the patient if they have coagulation disorders (such as taking anticoagulant drugs), diabetes (skin prone to infection), etc. If there is coagulation abnormality, prepare gauze for hemostasis in advance (the compression time should be extended to 8-10 minutes).

2. Procedure for Collecting Blood Culture Samples

(1) Aseptic operation and site disinfection

1) Environmental preparation: Place a disposable treatment towel beside the patient's bed in the general ward. Close the ward doors and windows (to prevent air circulation and contamination). Wipe the operation table surface with a chlorine-containing disinfectant, then the nurse washes her hands and puts on sterile gloves.

2) Disinfection of the venous puncture site:

Step 1: Dip a cotton swab in 2% chlorhexidine gluconate - 70% isopropyl alcohol compound disinfectant, and wipe the puncture point in a spiral pattern from the inside out, with a wiping range diameter of ≥ 8 cm;

Step 2: Maintain the wiping action for 30 seconds to ensure that the disinfectant fully penetrates the skin, then let it air dry (about 1-2 minutes). During this period, do not touch the disinfection area or wipe with sterile gauze.

(2) Blood collection and inoculation into the culture bottle

1) Venous puncture and blood collection:

① Tie a tourniquet (with the tightness suitable for being able to feel the arterial pulse), instruct the patient to clench their fists to fill the vein, hold the syringe at a 15-30° angle to insert, see the return blood, then fix the needle, slowly draw 20 mL of blood (avoiding excessive negative pressure that causes red blood cell rupture);

② After blood collection is completed, first loosen the tourniquet, then instruct the patient to loosen their fists, use sterile gauze to press the puncture point, remove the needle and continue to press (for routine patients, 5 minutes, for patients with abnormal blood coagulation, 8-10 minutes).

2) Inoculation into the culture bottle:

Unscrew the sealing caps of the BACTEC aerobic and anaerobic culture bottles, disinfect the surface of the bottle stopper with a 70% ethanol cotton ball, let it dry, then inject the blood into the two bottles separately, 10 mL each (strictly follow the sequence of "first anaerobic bottle, then aerobic bottle" to avoid air entering the anaerobic bottle and affecting bacterial growth);

After inoculation is completed, close the sealing caps of the culture bottles, hold the bottles with both hands and gently shake them 5-10 times (to allow the blood to fully contact the culture medium and prevent blood from clotting), avoid vigorous shaking

that causes the culture medium to spill.

(3) Sample Identification and Transportation

1) Sample Identification: Clearly fill in the patient's name, hospital number, collection date, collection time, puncture site (e.g. "left elbow median vein"), collector's name on the label of the culture bottle to ensure the information is consistent with the medical record.

2) Immediate Transportation:

Place the inoculated culture bottles in a biosafety transport box pre-tempered to 20-25℃. Avoid violent vibration during transportation;

Send to the clinical microbiology laboratory within 30 minutes and verify the sample information with the laboratory receiving personnel. Fill out the "Blood Culture Sample Submission Registration Form" and complete the handover procedures.

2. Bone marrow puncture

(1) Sampling frequency

This bone marrow puncture is a single collection procedure. Based on the diagnostic needs of the patient's condition, after the patient and their family sign the informed consent form, all the samples required for the tests are collected at one time. There is no repetition of the collection plan (if re-examination is needed in the future, it will be re-evaluated and a new collection plan will be formulated according to the progress of the patient's condition).

(2) Collection Site

The collection operation is carried out in the clinical diagnosis and treatment ward of the hematology department. An appropriate area beside the patient's bed is selected (ensuring sufficient operation space and a relatively enclosed environment), which is convenient for the patient to assume the right lateral position to cooperate with the puncture, and also allows for timely handling of any unexpected situations that may occur during the procedure.

(3) Sample size

Extract a small amount of bone marrow fluid (approximately 0.2 - 0.5 mL), depending on the requirement for preparing the smear.

(4) Collection Methods

1) Positioning: Assist the patient to assume a right lateral position, adjust the body posture to fully expose the left iliac posterior superior spine (the waist is slightly bent, the left lower limb is bent and flexed, and the right lower limb is extended, reducing the tension at the puncture site);

2) Puncture Point Location: Use the left iliac posterior superior spine as the puncture point (this area has thin bone, a large bone marrow cavity, and few blood vessels and nerves, making it a commonly used site for bone marrow puncture in clinical practice), mark the puncture center point on the skin surface with a marker pen;

3) Local Anesthesia: Use the layer-by-layer infiltration anesthesia method. Draw 5ml of 2% lidocaine injection solution. First, inject it on the skin surface of the puncture point to form a wheal, then slowly insert the needle along the puncture direction. Successively anesthetize the subcutaneous tissue, fascia, and periosteum. For each 0.5-1cm of insertion, inject a small amount of anesthetic to ensure that the anesthesia range covers the puncture path until the patient has no obvious pain (satisfactory anesthesia standard: the patient reports no stabbing or distending pain at the puncture point and its surroundings);

4) Puncture Operation:

a. The operator holds the bone marrow puncture needle, aligns the needle tip with the puncture point, and slowly rotates the needle vertically towards the bone surface (rotate evenly to avoid violent puncture that may cause bone damage). When the needle tip breaks through the bone cortex and enters the marrow cavity, a sudden decrease in resistance can be felt;

b. Remove the needle core, connect a 50ml syringe, and slowly draw a small amount of bone marrow fluid for smear (during the drawing process, be gentle to avoid excessive negative pressure that may dilute the bone marrow fluid or damage the cells);

c. After collecting the bone marrow fluid, connect the puncture needle connector, insert the needle core back, continue to rotate the needle slightly to make the needle tip enter the bone marrow substance, then remove the needle core, and use the cutting effect of the puncture needle to obtain bone marrow tissue;

5) Sample Processing: Immediately send the bone marrow fluid for examination after collection (smear samples are made on-site, while other liquid samples are injected into special anticoagulation tubes; bone marrow tissue samples are quickly placed in formalin solution for fixation to prevent tissue decay and deterioration).

(5) Tools used

1) Basic operation tools: sterile gloves, sterile gauze (size 30cm×30cm), disinfectant cotton balls, 2% lidocaine injection (5ml per vial), 5ml syringe (for anesthesia), 50ml syringe (for extracting bone marrow fluid);

2) Specialized puncture tools: disposable bone marrow puncture needle, puncture needle adapter;

3) Sample preservation tools: formalin fixative solution, slide for smear examination.

(6) Preoperative preventive measures

Informed consent and risk management: Clearly explain to the patient and their family the necessity of bone marrow puncture (for diagnosing the disease), the operation procedure, and possible unexpected situations (such as local bleeding, pain, infection, anesthesia allergy, etc.) after they have fully understood and signed the "Informed Consent Form for Bone Marrow Puncture". Only then can the operation be carried out.

(7) Post-collection processing and precautions

1) Care of the puncture site: After the procedure is completed, remove the bone marrow puncture needle and immediately apply sterile gauze to press the puncture site for 5-10 minutes (with moderate pressure, aiming to avoid bleeding). Once the bleeding stops, disinfect the puncture site again with iodophor and cover it with a sterile dressing (using breathable medical gauze and fixing it with adhesive tape). Inform the patient to keep the dressing on the puncture site dry for 24 hours, avoiding contact with water and scratching, and preventing infection;

2) Patient observation: After the procedure, observe the patient for 30 minutes, closely monitoring for symptoms such as dizziness, pale complexion, palpitations (excluding needle shock reactions), increased bleeding at the puncture site, swelling, or increased pain. If any abnormalities occur, handle them promptly;

3) Sample transportation: Place the collected bone marrow smear samples in a dedicated specimen transport box, label the patient's name, hospital number, test items, and collection time. Have a dedicated person deliver the samples to the corresponding testing departments in the laboratory and pathology departments within 30 minutes. During the handover, check the sample information and sign to confirm if there are no errors.
